# Determination of *RET* Sequence Variation in an MEN2 Unaffected Cohort Using Multiple-Sample Pooling and Next-Generation Sequencing

**DOI:** 10.1155/2012/318232

**Published:** 2012-04-01

**Authors:** R. L. Margraf, J. D. Durtschi, J. E. Stephens, M. Perez, K. V. Voelkerding

**Affiliations:** ^1^Research & Development, ARUP Institute for Clinical and Experimental Pathology, 500 Chipeta Way, Salt Lake City, UT 84108, USA; ^2^Department of Pathology, University of Utah School of Medicine, Salt Lake City, UT 84112, USA

## Abstract

Multisample, nonindexed pooling combined with next-generation sequencing (NGS) was used to discover *RET* proto-oncogene sequence variation within a cohort known to be unaffected by multiple endocrine neoplasia type 2 (MEN2). DNA samples (113 Caucasians, 23 persons of other ethnicities) were amplified for *RET* intron 9 to intron 16 and then divided into 5 pools of <30 samples each before library prep and NGS. Two controls were included in this study, a single sample and a pool of 50 samples that had been previously sequenced by the same NGS methods. All 59 variants previously detected in the 50-pool control were present. Of the 61 variants detected in the unaffected cohort, 20 variants were novel changes. Several variants were validated by high-resolution melting analysis and Sanger sequencing, and their allelic frequencies correlated well with those determined by NGS. The results from this unaffected cohort will be added to the *RET* MEN2 database.

## 1. Introduction

Multiple endocrine neoplasia type 2 (MEN2) is a rare autosomal dominant inherited disorder with a high lifetime risk of medullary thyroid carcinoma (MTC) [1, 2]. MEN2 consists of three syndromes: familial medullary thyroid carcinoma (FMTC), MEN2A, and MEN2B [1, 3]. FMTC families have only MTC. MEN2A families have MTC, with at least one individual developing pheochromocytomas, parathyroid hyperplasia, or both. MEN2B patients have MTC (with or without pheochromocytoma) and other characteristic clinical features: mucosal ganglioneuromas, GI ganglioneuromas, eye abnormalities, and skeletal abnormalities including marfanoid body habitus [4–7]. MEN2 is caused by pathogenic mutations found exclusively within the *RET* proto-oncogene (REarranged during Transfection). These are gain-of-function dominant mutations which are commonly heterozygous missense mutations found at specific codons within *RET* exons 10, 11, and 13–16 and rarely found within exons 5 and 8 [1, 8–10]. The medical management for the patient and potentially their family members is based on the familial *RET* variation, which is usually determined by Sanger sequencing [1]. Discovery of a known MEN2 pathogenic *RET* mutation within a family leads to screening for MTC, pheochromocytomas, or parathyroid hyperplasia, and potentially prophylactic thyroidectomy to increase survival rate for the intractable, aggressive MTC. Approximately 75–80% of MTC patients have the sporadic form of MTC (i.e., isolated, nonfamilial MTC), not MEN2 [6]. Patients with apparent sporadic MTC are always tested for an *RET* germline mutation, in case they actually have MEN2 and require different medical management. Although there are many well-known pathogenic *RET* mutations causative of MEN2, it may be difficult to know if a rare or novel germline *RET* variant is a pathogenic mutation (patient has MEN2) or nonpathogenic polymorphism (patient has sporadic MTC).

 Interpretation of rare and novel variants will increase in importance as more people are sequenced at the exome, whole genome, or targeted gene levels. Many new changes will be found with unknown clinical significance and their presence and allele frequency within the general population is of importance to help determine pathogenicity status of a variant. Consortiums like the 1000 genome and other large sequencing projects are making great progress in understanding population sequence variation. Yet more direct studies on single genes or gene panels can yield higher sequencing read coverage and more cost-effective sequencing over a smaller genetic area. Also, a particular chosen cohort can be sequenced for a particular locus, such as in the case of this study, where a cohort that was self-reported to have no personal or family history of MEN2 or MTC was sequenced for a section of the *RET* protooncogene where most pathogenic MEN2 causative mutations are located. *RET* sequence variation detected in this MEN2 unaffected population can then be added to the MEN2 *RET* database [8]. This data could be used for several reasons: (1) to help interpret the pathogenicity of clinically detected *RET* sequence variation; (2) as a reference for any future MEN2 case studies (variant was not found in those unaffected by MEN2 disease); (3) for improved genetic test design, to avoid or minimize designing probes or primers over known *RET* sequence variation.

To further reduce costs of sequencing large numbers of individuals, multiple samples can be pooled (without indexing) before next-generation sequencing (NGS). This was the focus of several studies that analyzed the ability to detect true variants within nonindexed pooled sample sets [11–16]. Thirty samples (60 alleles) were the maximum pooling number indicated by our prior studies and in other reports [12, 14, 17, 18], for reproducible and accurate singleton allele detection within the pool (a singleton is a unique allele within the pool). A pool of this size was expected to a have a singleton allele read frequency of 1.67%, and with consideration of sequencing error rates and potential variance in NGS determined variant read frequencies, singleton variants are expected to be detected above a cutoff of >1% variant reads [17, 18].

In this study, 136 individuals of an MEN2 unaffected cohort were sequenced on the illumina genome analyzer utilizing laboratory and bioinformatics protocols from our previous studies for nonindexed, multiple sample pooling. The pool size was limited to less than 30, which is the previously determined optimal pooling size for accurate singleton variant detection [12, 14, 17, 18]. In total, 61 variants were detected within the MEN2 unaffected cohort, which included 20 novel variants.

## 2. Materials and Method

### 2.1. Samples

Peripheral blood samples from 136 adult volunteers (113 Caucasian and 23 non-Caucasians for ethnic diversity) were collected and deidentified using University of Utah IRB protocol no. 7740. The donors for this unaffected cohort were self-described as not having a personal or family history of neither medullary thyroid carcinoma nor multiple endocrine neoplasia type 2 (MEN2). The 51 samples used as controls were deidentified according to IRB no.7275 and were Sanger sequenced for *RET* exons 10, 11, and 13–16, including exon/intron boundaries. The “single-sample control” did not have *RET* mutations causative of MEN2, while the “50 pool” control contained many samples with known MEN2 causative *RET* mutations. The 50 pool control was sequenced on the illumina genome analyzer several times previously [17, 18].

### 2.2. PCR, Library Prep, and NGS

DNA samples were amplified from *RET* intron 9 to intron 16 using long-range PCR technology. Amplicons were normalized by SequalPrep (Invitrogen Corp, Carlsbad, CA), quantified using Quant-iT Picogreen dsDNA kit (Invitrogen Corp), and equimolar pooled before Illumina Library Prep, utilizing previously described protocols [18]. Between 27 and 29 Caucasian samples' amplicons were combined into four separate pools (P1, P2, P3, and P4) before Illumina Library Prep and NGS. The non-Caucasian cohort's 23 samples were sequenced in a separate pool (ethnic pool). The PCR-amplified *RET* positions 1–9180 are positions 43608691–43617870 in reference sequence NC_000010.10 (Table 1). Two controls were also included in this study, a single sample and also a pool of 50 samples. Each pool and each control were sequenced in a separate flow cell lane on the illumina genome analyzer, using single-end read chemistry.

### 2.3. Data Analysis

Sequencing image files were processed and reads aligned to the *RET* reference sequence with SeqMan NGen version 2.1 software (DNAstar, Madison, WI), as described previously [17, 18]. Reads used were of 67 base lengths since the 3′ end read positions of longer reads can have an increase in sequencing background errors, as shown in previous studies [17, 19, 20]. As previously described, several base quality score screening thresholds (Q-threshold) evaluated for read coverage, errors (especially for outlier errors, which could be mistaken for false positives in a pool), variant read percentage, and base quality score statistics to determine the 30 Q-threshold should be used for analysis of all data sets, which minimized errors while maintaining adequate target read coverage (data not shown) [17, 18].

Excluded from analysis was a region of repeats and homopolymers that caused misalignment errors in all data sets (designated “repeat region,” amplicon positions 7686 to 7720). Changes from the reference sequence were designated variants, and the variant read percentage is the NGS-determined allele frequency. The previously developed subtractive correction method of variant detection was applied wherein the control's variant read percentages (at every position and possible variant change) are subtracted from the pooled data's variant read percentages, to yield a pooled data set without background sequencing error [17, 18].

### 2.4. Variant Validation

A subset of the NGS-detected *RET* sequence variants were validated by either high-resolution melting analysis (HRM) and/or Sanger sequencing. The HRM analysis PCR primers for *RET* exons 13 and 15 were described previously [21]. *RET* intron 9 used HRM analysis primers (5′ to 3′): forward ACA CTG CAA TGT GCG GGT CA and reverse GTC CCC CAA CAA TGC TGC CC. Sample DNA (~5 to 15 ng/uL final concentration) was amplified and analyzed as described previously [21], except the LightScanner 32 instrument (Idaho Technology, Inc., Salt Lake City, UT) which was used for both PCR and HRM analysis. The LightScanner parameters included Uracil-DNA glycosylase step (50°C for 10 min); polymerase activation (95°C for 10 min); 40 PCR cycles (denaturation at 95°C for 1 s, annealing at 62°C for 1 s, extension at 72°C for 4 s); formation of amplicon heteroduplexes (95°C for 1 s, then cool rapidly to 40°C for 10 s with ramp rate of 20°C/s); high-resolution melting protocol (70 to 96°C with ramp rate of 0.3°C/s) [21]. In order to detect samples with a homozygous *RET* variant that could not be distinguished from homozygous wild-type samples during HRM analysis, the same procedure was performed, except wild-type DNA (~5 ng/uL final concentration) which was spiked into the PCR reaction [22]. If needed, Sanger sequencing was used to confirm HRM determined variant results.

## 3. Results

### 3.1. Next-Generation Sequencing (NGS) of the 50-Pool and Single-Sample Controls

**Figure 1:**
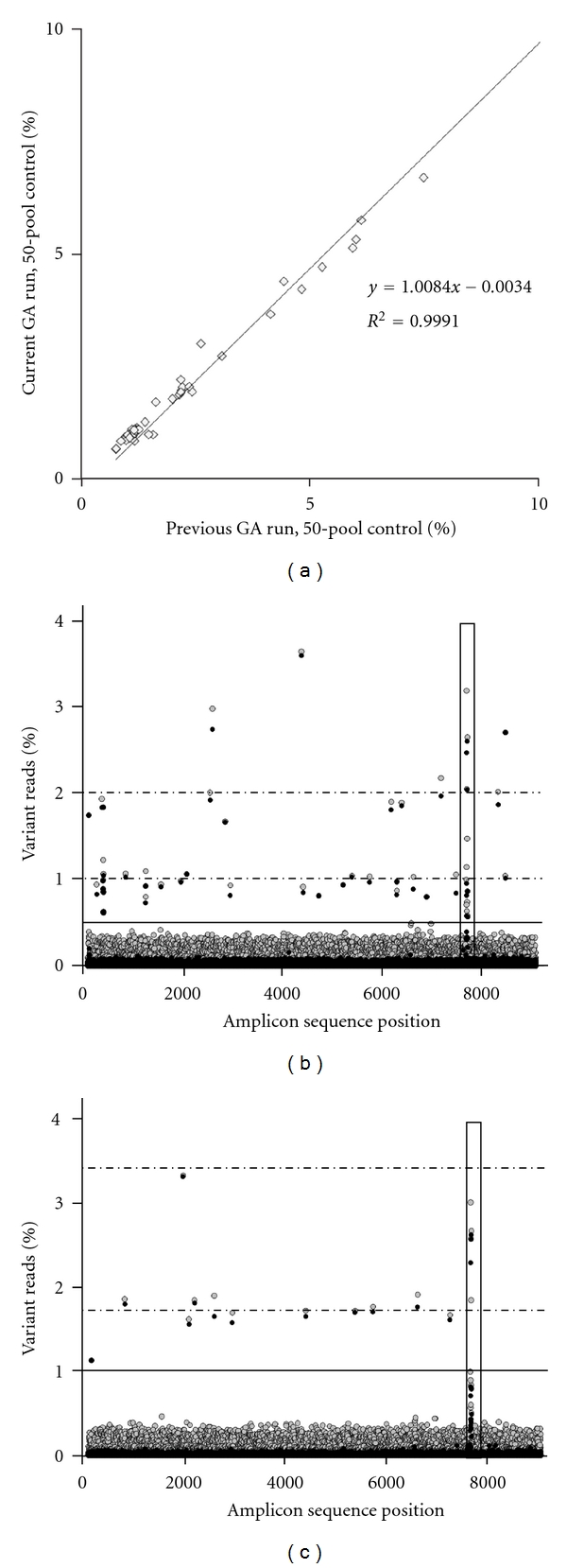
Variant identification in the 50-pool control and Caucasian P2 pool. (a) Variant read percentages for variants detected in the 50-pool control below 10% variant reads are shown. The variants detected in the 50-pool data for the current NGS run (*Y*-axis) is compared to the same library sequenced previously in a different NGS run (*X*-axis) with trendline and *R* ^2^ value shown on chart. (b and c) Variant read percentages for the pool data (gray circles) and the variant read percentages for the pool data after the subtractive correction with the single-sample control data (black circles) are shown together in each panel. The “repeat region” is boxed in black line. (b) 50-pool control data. Variant detection read cutoff value of 0.5% is the solid horizontal black line. The horizontal dotted lines mark the singleton and doubleton alleles' expected variant read percentages of 1% and 2%, respectively. (c) Caucasian pool P2 data. Variant detection read cutoff value of 1% is the solid horizontal black line. The horizontal dotted lines mark the singleton and doubleton alleles' expected variant read percentages of 1.7% and 3.4%, respectively.

The sequence of the single-sample control used in this study exactly matched the reference sequence and therefore had no true variant changes from the reference sequence, only background sequencing error (Table 1). This sample was an ideal control for error rates since any variant reads from the *RET* reference at each sequence position reflects the background NGS error rates, and also illumina genome analyzer sequencing has demonstrated reproducible, nonuniform, sequence-specific background error rates, read coverage, and base quality scores between lanes and runs using the same version chemistry [11, 12, 17–20, 23, 24]. This single sample controls for the sequence-specific error rates within the pooled data sets by using the subtractive correction method, as described in our previous studies [17, 18]. For subtractive correction, the single-sample control's variant reads at every possible sequence position, and change from the reference sequence is subtracted from the pool's variant read percentages. This yields an estimation of the pooled data without background sequencing error rates contributing to the variant read percentages (examples in Figure 1). The single-sample control and 50-pool data were also used for selection of the 30 Q-threshold used for quality screening of the data before analysis (data not shown) [17, 18].

 The 50-pool control demonstrated sensitivity to detect known variants with low read percentages for this NGS run and for using the subtractive correction method (as shown in Figure 1). The 50-pool contained 100 alleles and had an expected 1% singleton variant read frequency (singleton is unique within the pool). All 59 variants previously detected in the 50-pool were present at >0.5% variant reads and at similar percentage variant read values as determined in a previous NGS run with the same library (*R* ^2^ = 0.9991, Figure 1(a)). The 50-pool data had some potential false positives around the cutoff of 0.5% variant reads but after subtractive correction with the single-sample control data, and all true variants were readily detected from the background error (Figure 1(b)).

### 3.2. NGS of MEN2 Unaffected Cohort

Based on our previous work and other studies, sample pools were restricted to 30 or less samples within each pool to result in a variant read percentage above 1%, the chosen cutoff for the most accurate singleton variant detection [12, 14, 17]. The 113 Caucasian samples were divided into 4 pools with less than 30 samples. Caucasian pool P1 had 27 samples, P2 had 29, P3 had 28, and P4 had 29 samples, with an expected 1.85%, 1.72%, 1.77%, and 1.72% singleton variant read frequency, respectively. All pool data sets were evaluated with and without subtractive correction of sequencing background error rates using the single-sample control (Figure 1(c) and data not shown). A total of 51 variants were detected in the Caucasian MEN2 unaffected cohort with >1% variant read values, of which 23 were not found in the non-Caucasian MEN2 unaffected cohort (ethnic pool) (Table 1). The lowest singleton variant in the Caucasian data sets was in P2 with 1.12% variant read frequency. The 23 non-Caucasian samples were in one pool (ethnic pool), with an expected 2.17% singleton allele read frequency. A total of 38 variants were detected in this ethnically diverse MEN2 unaffected cohort with >1% variant read values, of which 10 were not found in the Caucasian data sets. The lowest singleton variant in the ethnic pool had 1.79% variant read frequency. The variant read percentages for each detected variant is shown in Table 1 per pool and also summarized for the four Caucasian pools. For comparison, the NCBI dbSNP allele frequency values for detected variants are also shown. All variants detected were intronic changes, except the expected common polymorphisms found in exons 11, 13, 14, and 15. Of the total 61 variants found in the MEN2 unaffected cohort, 20 variants were novel changes, not seen in the 50-pool control or in NCBI dbSNP132.

### 3.3. Validation of NGS-Detected Variations

**Figure 2:**
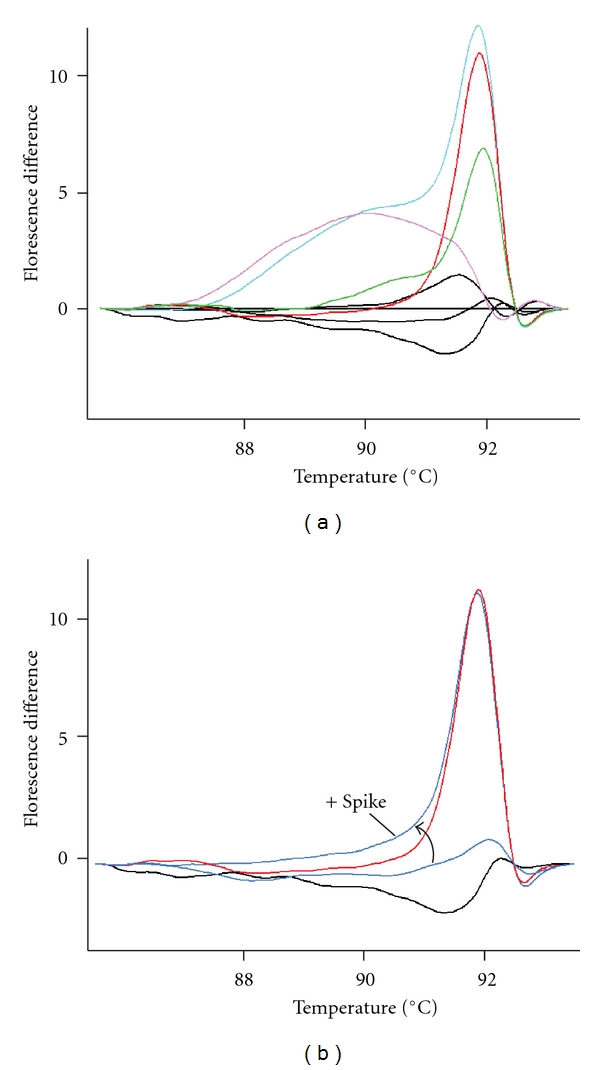
Variant validation within *RET* intron 9 by high-resolution melting analysis. The fluorescence difference plot (fluorescence difference versus temperature) of the melting curve data is shown in each panel. (a) *RET* intron 9. The black lines are samples of homozygous wild-type sequence. Data from four samples with unique variants within intron 9 are shown: heterozygous at amplicon position 156 (green trace, c.1760−158C>G), heterozygous at 174 (red trace, c.1760−140C>G), heterozygous at 117 (pink trace, c.1760−197G>T), and a sample heterozygous at both positions 117 and 174 (light blue). (b) Intron 9 with wild-type DNA spiked into the PCR reaction to help differentiate homozygous variants. One sample with a homozygous variant at position 174 with (“+spike”) and without wild-type DNA spiked in is shown (blue traces) compared to a 174 heterozygous (red trace) and wild-type sample (black trace).

Since the 136 unaffected cohort samples had not been sequenced previously by either Sanger or NGS methods, several variant locations within three pools (total of 79 samples) were chosen for validation. The high-resolution melting (HRM) analysis method, which is a rapid, closed-tube mutation scanning assay, was chosen to genotype each individual sample for validation of NGS variant detection and the NGS determined variant allele frequency (Table 2). High-resolution melting analysis detects sequence variation within the PCR amplicon using a saturating dsDNA dye and in many cases can uniquely identify each variant based on differential melting profiles (Figure 2(a)) [25–28]. HRM assay states 100% specificity and sensitivity for detection of heterozygous variants within small amplicons (<300 bp) [29]. HRM analysis was used to detect sequence variations within a section of *RET* intron 9 and exons 13 and 15 (Figure 2 and data not shown). *RET* exons 13 and 15 were chosen since they each contain a common polymorphism present in all pools that could be detected using the previously developed HRM assay [21]. Exon 13 contains c.2307G>T variant and exon 15 contains c.2712C>G variant (*RET* amplicon positions 5153 and 6943, respectively, Tables 1 and 2). Intron 9 was chosen since it contains three NGS-detected variants in close proximity at *RET* amplicon positions 117, 156, and 174 (c.1760−197G>T, c.1760−158C>G, and c.1760−140C>G, resp.), and also to verify the novel c.1760−158C>G variant detected in Caucasian pool P2 which had the lowest variant read percentage of 1.12% (expected 1.72% singleton read frequency within that pool of 29 samples). Since some homozygous variants can have similar melting profiles as the wild-type sample [22], a technique that spikes wild-type DNA into the PCR reaction to allow distinction of homozygous variants was performed on any sample that appeared wild-type after testing in the first HRM assay. This technique identified four homozygous variants in *RET* exon 15 and one homozygous variant in *RET* intron 9 (Figure 2(b) and data not shown). The HRM determined allele frequency correlated well with the NGS variant read percentage for each variant in each pool (Table 2). The variant with the lowest read percentage (position 156 in P2, 1.12%) was verified as present and heterozygous in one sample within Caucasian pool P2.

## 4. Discussion

This paper describes *RET* proto-oncogene sequence variation detected in an MEN2 unaffected cohort of 136 individuals. The previous genome analyzer sequenced 50-pool library [17, 18] was used to control for the detection of variations with low read frequency, and all known variants were detected >0.5% variant reads. With similar error rates between genome analyzer lanes of the same run [11, 12, 17, 19, 20, 23, 24], the singleton variants in a less than or equal to 30-sample pool should be accurately detected above background error using our previously determined cutoff value of >1% variant read frequency. The single sample controlled for background sequencing error rates across each *RET* sequence position and was used for the subtractive correction method of variant detection for pools [17, 18].

The majority of the MEN2 unaffected cohort were of Caucasian ethnicity, while 23 samples were non-Caucasian (ethnic pool) and were used to identify *RET* variants within a more ethnically diverse sample set. The 136 samples were distributed into five nonindexed pools and were sequenced in five separate flow cell lanes. Using previously described protocols for bioinformatics, subtractive correction, and variant read cutoff value of >1% [17, 18], a total of 61 *RET* variants were detected within the MEN2 unaffected cohort. Twenty of these variants were novel, not in NCBI dbSNP 132 (which includes 1000 Genome data) or found in our previous sample pooling studies on the *RET* proto oncogene [17, 18]. Many of these novel changes were specific to either the Caucasian or ethnic samples and were of low variant read frequency, so they were likely to be singleton or doubleton variants within the pools. Several variants were verified by HRM and Sanger sequencing, including the novel change (c.1760−158C>G) with the lowest variant read percentage (1.12%).

The *RET* MEN2 database developed by the author so far has 147 entries, of which 74 are known pathogenic mutations and 62 are variants of uncertain significance. This database has been used as a model for predictions of phenotypic severity of variants of unknown clinical significance within the *RET* proto oncogene [30]. *RET* sequence variation data for these MEN2 unaffected cohorts will be added to the MEN2 *RET* database [8]. This variant data will help in medical interpretation of variations found in this *RET* proto-oncogene region using methods such as Sanger sequencing or NGS (targeted to the *RET* gene, the whole exome, or whole genome sequencing). Any *RET* sequence change detected in individuals with a family history of MEN2 symptoms or where MEN2 is suspected (patient with apparent sporadic MTC or Pheochromocytoma) can be compared to the available *RET* MEN2 database, and also to the benign *RET* sequence variation present in the large cohort of unaffected individuals that was generated in this paper. This highlights the importance of clinically relevant databases with not only known pathogenic changes, but also the inclusion of known benign changes for clinical test interpretation. The MEN2 unaffected cohort's variant results can also be used in comparison to variants detected in suspected MEN2 patients for case reports. A potential problem for genetic test design is unknown variants present in the location of the PCR primers, Sanger sequencing primers, or melting analysis probes. Results from this unaffected population will help with genetic test design, so that primers, and probes will not be designed over the known *RET* sequence changes.

This paper presents sequence variation detection methods that could be used for other genes and analysis for specific cohorts (unaffected versus affected, by different ethnicities, or those with specific symptoms of disease). The resulting data can be added to locus-specific databases to help interpret the pathogenicity of clinically detected sequence variation. These validated methods can also apply to other pooled samples (such as genetic locations for GWAS followup or for testing-specific populations) and natural pools (such as mitochondrial heteroplasmy or mixed tumor populations).

## Figures and Tables

**Table 1:** Variants detected in the pooled data sets and their NGS percent variant read values^a^.

RET amplicon position	Chr 10 positions	RET gene location	Genotype	dbSNP	dbSNP allele frequency	Found only in which pool	Ethnic	Caucasian combined pop freq	P1	P2	P3	P4	Control
							% variant reads		% variant reads	% variant reads	% variant reads	% variant reads	% variant reads
117	43,608,807	Intron 9	c.1760−197G>T	112675631	8.0%		6.20%	6.19%	5.62%	8.05%	7.77%	2.90%	0.00%
156	43,608,846	Intron 9	c.1760−158C>G	Novel		Caucasian	0.01%	0.88%	0.01%	1.12%	0.00%	1.49%	0.00%
174	43,608,864	Intron 9	c.1760−140C>G	3026758		Ethnic	6.66%	0.00%	0.00%	0.00%	0.00%	0.00%	0.00%
819	43,609,509	Intron 10	c.1880−419G>A	Novel		Caucasian	0.05%	0.44%	0.06%	1.85%	0.05%	0.05%	0.06%
1429	43,610,119	exon 11	c.2071G>A	1799939	14.7%		12.79%	18.14%	15.00%	15.33%	17.30%	24.78%	0.07%
1645	43,610,335	Intron 11	c.2136+151G>A	Novel		Caucasian	0.10%	0.44%	1.92%	0.10%	0.10%	0.10%	0.12%
1676	43,610,366	Intron 11	c.2136+182G>A	1864400	75.2%		68.62%	75.66%	79.53%	76.35%	68.79%	79.64%	0.02%
1765	43,610,455	Intron 11	c.2136+271T>C	1864399	73.2%		63.96%	69.47%	72.74%	67.72%	62.75%	75.90%	0.10%
1766	43,610,456	Intron 11	c.2136+272G>A	Novel		Caucasian	0.03%	0.44%	1.79%	0.04%	0.03%	0.03%	0.02%
1868	43,610,558	Intron 11	c.2136+374C>T	2742233	72.3%		61.79%	69.47%	73.34%	67.78%	61.11%	76.34%	0.07%
1931	43,610,621	Intron 11	c.2136+437T>C	Novel		Caucasian	0.16%	0.44%	0.15%	0.14%	2.15%	0.17%	0.13%
1981	43,610,671	Intron 11	c.2136+487G>T	3026762	6.2%		6.09%	9.29%	8.80%	3.32%	10.17%	13.42%	0.01%
2003	43,610,693	Intron 11	c.2136+509G>T	Novel		Caucasian	0.01%	0.44%	1.75%	0.00%	0.01%	0.01%	0.00%
2096	43,610,786	Intron 11	c.2136+602T>C	Novel		Caucasian	0.08%	0.88%	1.59%	1.61%	0.06%	0.07%	0.06%
2207	43,610,897	Intron 11	c.2136+713C>T	Novel		Caucasian	0.04%	0.44%	0.04%	1.84%	0.03%	0.03%	0.03%
2598	43,611,288	Intron 11	c.2137−744A>G	50 pool		Caucasian	0.23%	1.33%	1.94%	1.89%	0.22%	1.68%	0.24%
2618	43,611,308	Intron 11	c.2137−724G>A	Novel		Caucasian	0.07%	0.44%	1.92%	0.06%	0.07%	0.06%	0.05%
2958	43,611,648	Intron 11	c.2137−384C>T	50 pool			1.92%	0.44%	0.08%	1.69%	0.09%	0.09%	0.12%
3018	43,611,708	Intron 11	c.2137−324A>G	741968	76.0%		68.18%	76.11%	78.72%	76.37%	68.79%	81.20%	0.04%
3089	43,611,779	Intron 11	c.2137−253C>T	74135468	6.9%	Ethnic	1.54%	0.00%	0.03%	0.03%	0.04%	0.04%	0.04%
3175	43,611,865	Intron 11	c.2137−167T>C	2256550	47.2%		46.33%	45.58%	51.81%	45.88%	39.24%	45.15%	0.22%
3535	43,612,225	Intron 12	c.2284+46G>C	Novel		Ethnic	1.85%	0.00%	0.00%	0.00%	0.00%	0.01%	0.00%
3536	43,612,226	Intron 12	c.2284+47C>T	760466	14.5%		10.07%	17.26%	18.80%	15.39%	14.88%	20.45%	0.05%
3919	43,612,609	Intron 12	c.2284+430C>T	2742234	70.1%		62.23%	69.91%	73.95%	68.56%	60.98%	76.51%	0.04%
4382	43,613,072	Intron 12	c.2285−749C>T	3026765	2.2%	Caucasian	0.04%	4.87%	5.61%	6.82%	3.42%	3.62%	0.05%
4418	43,613,108	Intron 12	c.2285−713G>A	79045327	0.4%		1.97%	0.44%	0.06%	1.71%	0.06%	0.05%	0.07%
4503	43,613,193	Intron 12	c.2285−628T>C	Novel		Caucasian	0.23%	0.44%	1.99%	0.23%	0.23%	0.24%	0.25%
4710	43,613,400	Intron 12	c.2285−421G>A	114921735	2.9%	Ethnic	1.86%	0.00%	0.11%	0.08%	0.09%	0.08%	0.11%
4750	43,613,440	Intron 12	c.2285−381G>A	Novel		Ethnic	2.15%	0.00%	0.09%	0.08%	0.07%	0.09%	0.10%
5153	43,613,843	Exon 13	c.2307G>T	1800861	72.3%		62.24%	70.35%	74.10%	68.58%	61.27%	76.98%	0.01%
5397	43,614,087	Intron 13	c.2392+159G>A	3026767	1.6%	Caucasian	0.03%	3.54%	3.37%	1.71%	4.93%	3.68%	0.02%
5540	43,614,230	Intron 13	c.2392+302G>A	2075910	72.2%		62.45%	70.35%	74.57%	68.59%	61.28%	76.56%	0.04%
5630	43,614,320	Intron 13	c.2392+392G>A	Novel		Caucasian	0.04%	0.44%	0.03%	0.03%	0.02%	1.60%	0.03%
5752	43,614,442	Intron 13	c.2392+514G>A	50 pool		Caucasian	0.06%	0.88%	0.06%	1.76%	1.93%	0.06%	0.07%
5770	43,614,460	Intron 13	c.2393−519G>A	2075911	14.7%		12.43%	18.14%	14.55%	14.88%	17.04%	24.21%	0.04%
5820	43,614,510	Intron 13	c.2393−469C>A	Novel		Caucasian	0.02%	0.44%	1.82%	0.01%	0.01%	0.01%	0.01%
6004	43,614,694	Intron 13	c.2393−285G>A	78453984	5.4%	Caucasian	0.03%	0.44%	0.03%	0.02%	0.03%	1.52%	0.03%
6195	43,614,885	Intron 13	c.2393−94C>T	111264957	4.2%		5.77%	6.19%	5.22%	7.80%	7.37%	3.01%	0.09%
6221	43,614,911	Intron 13	c.2393−68A>G	Novel		Ethnic	2.19%	0.00%	0.18%	0.15%	0.15%	0.15%	0.12%
6404	43,615,094	exon 14	c.2508C>T	1800862	4.1%		5.86%	5.75%	5.13%	7.67%	7.39%	2.98%	0.04%
6639	43,615,329	Intron 14	c.2607+136A>G	50 pool			2.06%	0.44%	0.16%	1.90%	0.15%	0.17%	0.14%
6692	43,615,382	Intron 14	c.2608−147C>T	11238441	14.8%		12.78%	18.58%	14.55%	15.09%	17.30%	25.23%	0.06%
6696	43,615,386	Intron 14	c.2608−143C>G	Novel		Ethnic	1.90%	0.00%	0.00%	0.00%	0.00%	0.00%	0.00%
6715	43,615,405	Intron 14	c.2608−124G>A	111306965	8.9%	Ethnic	1.93%	0.00%	0.04%	0.06%	0.04%	0.05%	0.06%
6815	43,615,505	Intron 14	c.2608−24G>A	2472737	16.0%		19.84%	17.26%	21.52%	18.75%	14.39%	13.81%	0.04%
6943	43,615,633	Exon 15	c.2712C>G	1800863	14.9%		11.74%	16.37%	13.17%	13.92%	16.24%	22.34%	0.00%
7190	43,615,880	Intron 15	c.2730+229T>C	3026768	5.1%		6.07%	6.64%	5.40%	8.07%	7.75%	4.57%	0.21%
7218	43,615,908	Intron 15	c.2730+257C>T	2435353	14.4%		19.91%	17.26%	21.81%	18.89%	14.51%	14.19%	0.08%
7283	43,615,973	Intron 15	c.2730+322C>T	3026769	4.3%		1.79%	1.33%	0.07%	1.66%	1.68%	1.37%	0.06%
7392	43,616,082	Intron 15	c.2730+431G>A	Novel		Ethnic	1.93%	0.00%	0.17%	0.13%	0.17%	0.17%	0.18%
7491	43,616,181	Intron 15	c.2730+530A>G	79094522	11.1%	Caucasian	0.21%	0.44%	0.25%	0.23%	0.20%	1.66%	0.22%
7635	43,616,325	Intron 15	c.2730+674A>G	2742235	76.0%		68.78%	76.99%	79.66%	77.02%	71.18%	79.24%	0.18%
7844	43,616,534	Intron 15	c.2731−860G>A	Novel		Ethnic	1.94%	0.00%	0.02%	0.03%	0.02%	0.03%	0.02%
8061	43,616,751	Intron 15	c.2731−643C>A	715106	76.7%		68.41%	77.43%	79.60%	77.02%	70.90%	80.13%	0.01%
8179	43,616,869	Intron 15	c.2731−525G>A	Novel		Caucasian	0.04%	0.44%	1.80%	0.03%	0.03%	0.04%	0.03%
8181	43,616,871	Intron 15	c.2731−523T>G	Novel		Caucasian	0.01%	0.88%	0.00%	0.00%	0.01%	3.48%	0.00%
8328	43,617,018	Intron 15	c.2731−376A>G	3026770	1.0%	Caucasian	0.17%	0.88%	0.17%	0.17%	1.81%	1.47%	0.15%
8414	43,617,104	Intron 15	c.2731−290A>G	2565202	47.6%		47.17%	52.21%	58.83%	52.76%	45.68%	50.20%	0.05%
8477	43,617,167	Intron 15	c.2731−227C>G	3026771	4.5%		5.82%	6.19%	5.09%	7.64%	7.36%	4.31%	0.00%
8828	43,617,518	intron 16	c.2801+54A>T	3026772	0.5%	Caucasian	0.01%	0.44%	0.00%	0.00%	0.00%	1.66%	0.00%
9017	43,617,707	intron 16	c.2801+243G>C	3026774	2.1%	Caucasian	0.02%	5.75%	5.21%	6.66%	6.77%	3.63%	0.00%

^a^Table headings. *RET* amplicon position and Chr 10: amplicon positions 1–9180 correlate to Chr 10 positions 43608691–43617870 in reference sequence NC_000010.10. Genotype: nomenclature (cDNA) for known variants is from the MEN2 RET database—http://www.arup.utah.edu/database/MEN2/MEN2_welcome.php, which uses the Human Genome Variation Society sequence variation nomenclature and RET reference sequence NC_000010.10. dbSNP column lists rs number, “50 pool” (if novel from dbSNP, but was found in 50 pool data set), or “novel” change. Found only in which pool: variation was found only within the ethnic or Caucasian pools. % variant reads: NGS determined variant read percentage for each variant found within each pool. Ethnic: data was from 23 pooled non-Caucasian samples. Caucasian combined pop freq: it combines all the data from the 4 Caucasian pools (P1, P2, P3, and P4), for comparison to the ethnic pool as well as the NCBI dbSNP 132 stated allele frequencies. Control: this single-sample control matched the reference sequence exactly, so no variations were detected and the values in this control column are only background sequencing error rates (0% to 0.25% variant read values).

**Table 2:** Validation of several variants and comparison of NGS and HRM determined variant allele frequencies.

Pool	No. of samples^a^	117^b^ in intron 9		156 in intron 9		174 in intron 9		5153 in exon 13		6943 in exon 15	
		NGS^c^	HRM^c^	NGS	HRM	NGS	HRM	NGS	HRM	NGS	HRM
Ethnic	23	6.2%	6.5%	0.01%	0.00%	6.7%	8.7%	62%	63%	12%	13%
P1	27	5.6%	5.6%	0.01%	0.00%	0.0%	0.0%	74%	74%	13%	19%
P2	29	8.1%	8.6%	1.12%^d^	1.70%	0.0%	0.0%	69%	72%	14%	16%

^a^A total of 79 samples were individually tested for variants by HRM analysis for three regions of the *RET* gene (which analyzed the 5 NGS-detected variant positions shown in this table).

^b^ *RET* amplicon position shown, see Table 1 for more information on each variant change.

^c^NGS: illumina genome analyzer determined allele frequency (variant read percentage) from a pooled sample set. HRM: high-resolution melting analysis determined allele frequency, where each individual in the pool was tested separately for variation.

^d^Lowest NGS variant read percentage for all pools. This suspected variant was verified as a singleton allele within Caucasian pool P2 by HRM and Sanger sequencing.

## Abbreviations

MEN2: Multiple endocrine neoplasia type 2
*RET*: REarranged during transfection
MTC: Medullary thyroid carcinoma
FMTC: Familial medullary thyroid carcinoma
PCR: Polymerase chain reaction
Q-threshold: Base quality score screening threshold
NGS: Next generation sequencing.
